# Pseudoaneurysm formation following core needle biopsy in a patient diagnosed with breast cancer: A case report

**DOI:** 10.1016/j.radcr.2024.08.106

**Published:** 2024-09-14

**Authors:** Reem Ahmed Al Mazrouai, Asma Al Shizawi, Badriya Al Qassabi, Suaad Al Aghbari

**Affiliations:** a Sultan Qaboos Comprehensive Cancer Care and Research Centre (SQCCCRC), Oman, Muscat; b Oman Medical Specialty Board (OMSB), Oman, Muscat

**Keywords:** Core needle biopsy, Breast pseudoaneurysms, Breast tumor, Breast ultrasound, Therapeutic management

## Abstract

Core needle biopsy is a common diagnostic procedure in breast cancer patients, but it can occasionally lead to serious complications. We report a rare case of pseudoaneurysm formation following a core needle biopsy in a 54-year-old female patient diagnosed with breast cancer. Despite the routine nature of the procedure, the patient developed a palpable mass at the biopsy site, which prompted further diagnostic imaging and interventions. The pseudoaneurysm was effectively treated using a percutaneous approach with ultrasound-guided thrombin injection, demonstrating a minimally invasive solution that promptly addressed the complication without the need for surgical intervention. This case highlights the critical importance of detecting complications early in the biopsy process, as they have significant implications for disease staging and treatment initiation. It also underscores the importance of being prepared for immediate intervention in case of biopsy-related complications like pseudoaneurysms, to prevent severe consequences.

## Introduction

Core needle biopsy (CNB) is the primary method for sampling potentially malignant breast masses, often assisted by ultrasound. This minimally invasive procedure is pivotal for presurgical assessment of histological grade, tumor subtype, and receptor status. CNB typically causes minimal complications, such as hematoma and bleeding, usually managed conservatively [1,2]. However, rare cases of iatrogenic pseudoaneurysms (PSA) may occur. These false aneurysms result from arterial wall breaks, leading to blood accumulation outside the vessel, opposite to true aneurysms which involves all vessel layers [3]. PSA in breast commonly arise from branches of 2 key arterial vessels: the internal thoracic artery, responsible for supplying blood to the medial and central regions of the breast, and the lateral thoracic artery, which serves the lateral regions [4,5].

Pseudoaneurysms, although rare, can be a serious complication, especially if they rupture, leading to significant hemorrhage. It's crucial for these conditions to be closely monitored and appropriately managed to mitigate risks [6].

In addition, the presence of a pseudoaneurysm in a breast cancer patient can complicate the clinical picture. If a pseudoaneurysm is misinterpreted as another tumor, it could lead to an inaccurate assessment of tumor size and stage, potentially affecting the choice of adjuvant therapy [7]. Additionally, initiating chemotherapy before resolving a pseudoaneurysm might exacerbate bleeding and increase the size of the aneurysmal sac. This can lead to further complications and might impact the evaluation of the tumor's response postchemotherapy [7,8].

We present a case involving a 51-year-old female who developed a breast pseudoaneurysm after undergoing an ultrasound-guided core breast biopsy at another institution. Upon seeking our center's expertise, it was noted that instead of a single mass, she presented with 2 masses.

Our objective is to highlight the importance of recognizing pseudoaneurysms and differentiating them from other breast masses. Furthermore, we stress the critical need for proper management before advancing to tumor staging and commencing chemotherapy. The literature currently lacks detailed exploration of iatrogenic PSAs in breast cancer patients, emphasizing the significance of this report.

## Case report

**Fig. 1:**
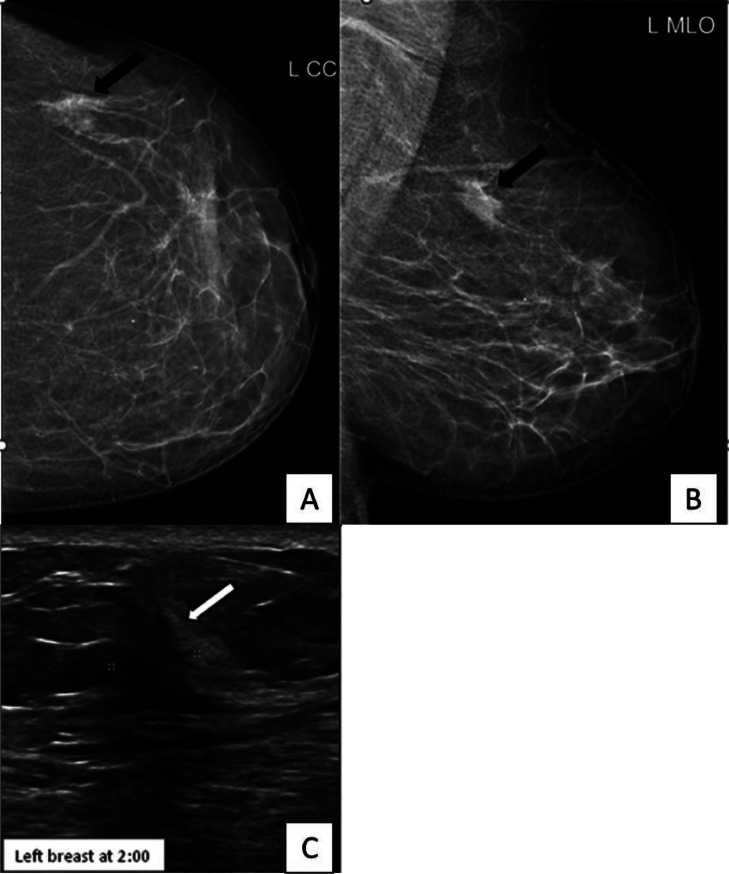
(Imaging of a breast lesion in the left upper outer quadrant). Image (A): Full paddle digital mammography craniocaudal view and image (B): Full paddle digital mediolateral oblique view, both demonstrating an irregular high-density mass at upper outer quadrant (black arrow) classified as BIRADS 5 according to ACR lexicon. Image (C): Axial grey scale ultrasound image of the same lesion shows corresponding irregular hypoechogenic mass at 2:00 measuring 1 cm (white arrow).

A 51-year-old woman presented to a community hospital with a left breast palpable lump. She underwent diagnostic mammography and Ultrasound (US) which revealed an irregular, speculated mass at the upper outer quadrant at 2 o'clock radian, measuring 0.8 × 1.3 × 1 cm. It was categorized based on Breast Imaging Reporting and Date System as BI-RADS 5 (suspicious finding) [9] (Fig. 1).

Consequently, core biopsy was performed with the patient's consent to obtain a definitive diagnosis. Biopsy was performed using a 14-gauge maxcore biopsy needle. The histopathological diagnosis of the core biopsy showed invasive lobular carcinoma, grade II, ER positive, PR positive and Her2 neu negative, Ki-67 20%. Fine needle aspiration of left axillary node was positive for metastasis.

Five weeks later, the patient was referred to oncology center for surgical oncological management.

At the time of presentation, 2 lumps were felt in the clinical examination by the breast surgeon at 2 o'clock and 1 o'clock associated with mild erythema of the overlying skin. Since there is discrepancy between clinical examination showing 2 masses while outside breast imaging reported a single mass, a repeat breast imaging in our center was recommended.

Ultrasound examination of the left breast showed an irregular hypoechoic mass with indistinct margins and posterior shadowing at 2 o'clock. It measured 0.8 × 1.3 × 1 cm (Fig. 1). This corresponds to the known biopsy proven malignancy (BIRADS 6).

**Fig. 2:**
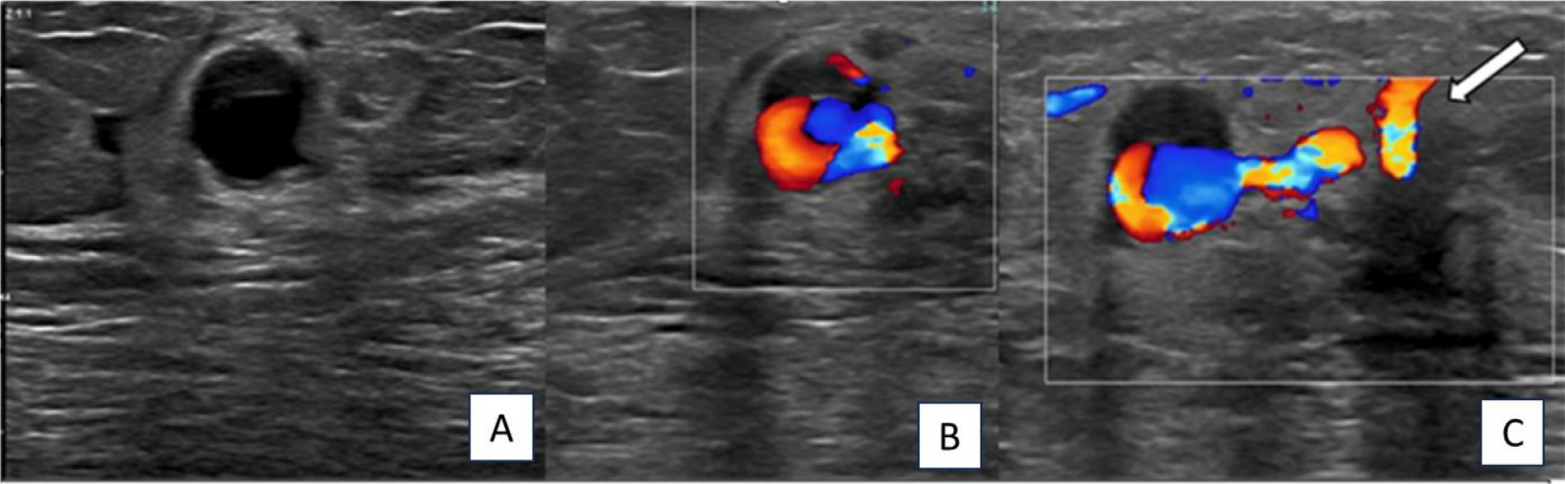
Axila greyscale ultrasound examination shows rounded anechoic mass at the site of prior biopsy area (image A), medial to the piopsy proven malignant mass. Color Doppler shows blood flow inside the mass with characteristic “yin-yan” sign of pseudo aneurysm (image B). Note associated adjacent artery (white arrow in image C).

Additionally, there was a round, well-defined, anechoic mass situated at the 1 o'clock position, just medial to the piopsy proven malignant mass. It measured approximately 0.7 × 0.6 × 0.6 mm (as shown in Fig. 2). Color Doppler sonography demonstrated turbulent flow within the mass, which was connected to an adjacent prominent blood vessel supplying the malignant mass through a narrow neck. The mass did not appear to be a new satellite cancer, but rather an iatrogenic pseudoanerusysm, presumably caused by a recent biopsy (Fig. 2).

Case was discussed in multidisciplinary meeting, and it was decided to start with neoadjuvant chemotherapy according to the disease stage and hormonal status. Considering the potential risk of rupture or bleeding from the adjacent pseudoaneurysm, pseudoaneurysm occlusion was recommended.

Following the MDT meeting's recommendations, the patient was called back to the clinic for counseling on treatment options. She opted for adjuvant therapy followed by surgical excision and sentinel lymph node biopsy. The oncologist and surgeon informed her about complications stemming from the previous biopsy, leading to a pseudoaneurysm. Stressing the need to address this issue before starting chemotherapy due to potential bleeding risks, various treatment options, including conservative techniques and surgical interventions, were discussed. However, the patient ultimately chose conservative management.

**Fig. 3:**
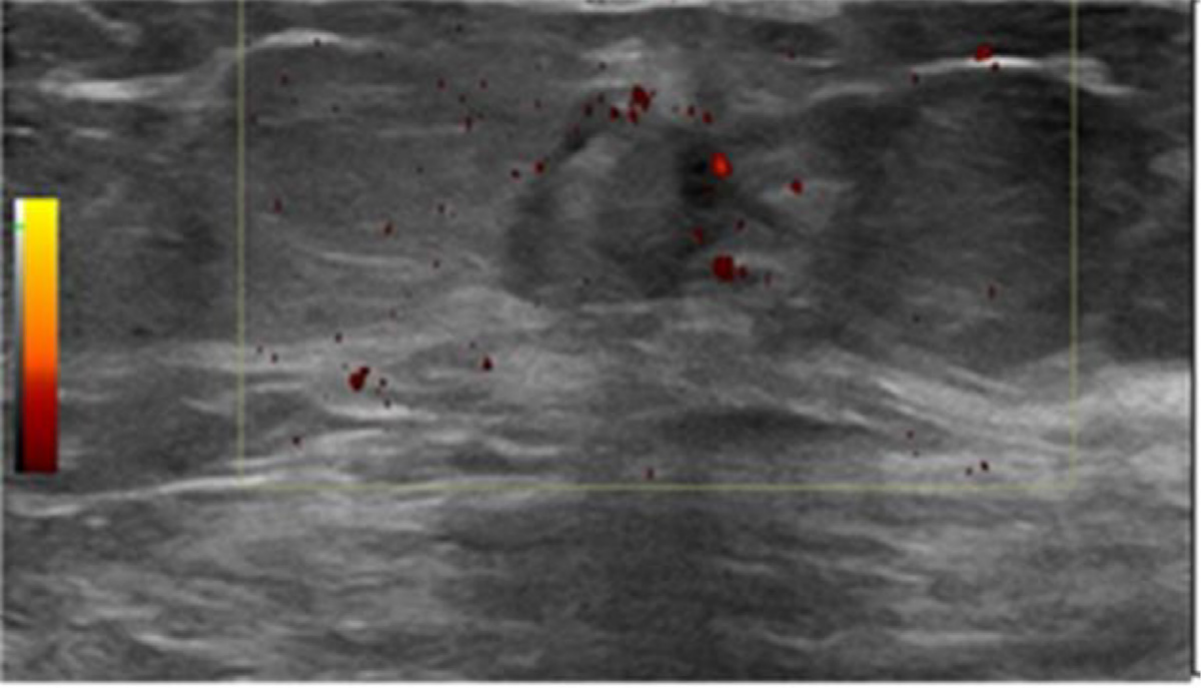
Follow-up ultrasound image after injection confirms pseudo aneurysm thrombosis with no color flow in color doppler study.

The patient was scheduled for treatment in the radiology department using ultrasound guidance. A conservative approach was started with local compression applied to the site of the left breast pseudoaneurysm for 30 minutes, followed by 20 minutes of sonographic-guided compression. However, repeated Doppler ultrasound showed persistent flow within it denoting failure of spontaneous thrombosis. After that patient was offered percutaneous treatment of the PSA using ultrasound guided thrombin injection. The procedure was conducted by an interventionist radiologist with surgeon attendance. Thrombin-JMI (5000 IU) was diluted with isotonic saline to yield 1 hundred international units per mL. The solution was then injected with a 23-gauge needle. Initially, 1 mL was injected into the PSA followed by another 1 mL injected into the connected stalk after a short interval. Hemostasis was achieved by direct compression. Recheck ultrasound and color Doppler showed complete thrombosis of the mass with no active blood space. The patient was hemodynamically stable during the procedure. The patient was discharged home with a follow up schedule in breast -surgery clinic. The follow-up breast sonogram after 3 months showed a thrombosed pseudoaneurysm that had regressed in size (Fig. 3).

## Discussion

Iatrogenic pseudoaneurysms in the breast, while rare, predominantly occur following core needle biopsy (CNB). Alternative causes include blunt breast trauma, a history of breast implants, and underlying malignancy [10]. Spontaneous pseudoaneurysms are exceedingly uncommon, with only few cases documented in literature. Risk factors like age, female gender, existing atherosclerosis, anticoagulant therapy, pregnancy, and lactation can increase the likelihood of pseudoaneurysm post-CNB [10].

Breast pseudoaneurysms can manifest through various symptoms such as a pulsatile mass, pain, swelling, bruising, and skin discoloration. Nonetheless, some cases remain asymptomatic, only identified incidentally during imaging procedures [11]. Color-flow Doppler ultrasound stands as the primary diagnostic tool, with more than 95% accuracy rate. The swirling, or yin-yang, flow pattern on color Doppler images and a to-and-fro waveform on spectral Doppler sonography are indicating the diagnosis of pseudoaneurysm [12,13]. Other imaging studies, such as angiography, may be used in some cases.

Treatment strategies for breast pseudoaneurysms are tailored based on the pseudoaneurysm's size and location, the patient's symptoms, and their overall health condition. These strategies can be organized into 3 main categories, conservative, minimal invasive procedures and surgical intervention [13]. In cases of small, asymptomatic pseudoaneurysms, conservative methods like observation and compression are often sufficient. These approaches involve monitoring the condition with minimal intervention. However, larger or symptomatic pseudoaneurysms may require more proactive treatment. This often involves minimally invasive procedures, such as injecting substances like alcohol, thrombin, or micro coils. These substances are considered safe and reasonable for use [13,14]. Endovascular management is another minimally invasive option. This technique includes direct puncture under ultrasound guidance, with treatments like coil embolization or thrombin (Factor IIa) injection. Both methods are effective in embolizing pseudoaneurysms and have been successfully used in patient treatment [14].

In cases where these methods are unsuccessful or unsuitable, surgical intervention may be considered. This more invasive approach typically involves locating and ligating the feeding artery of the pseudoaneurysm, and it may include resecting a malignant breast mass if present [15]. Despite the seriousness of these interventions, routine follow-up ultrasound is not commonly recommended, as such complications are rare and generally do not pose a significant health risk to the patient [13].

The prognosis for patients with iatrogenic breast pseudoaneurysms is generally favorable, provided there is timely diagnosis and appropriate treatment [13]. This case underscores the importance of considering pseudoaneurysm as a potential complication following breast interventions, particularly in individuals with risk factors, and highlights the effectiveness of tailored treatment approaches based on individual patient profiles.

## Conclusion

Pseudoaneurysm formation post-CNB, while rare, presents a clinical dilemma that requires prompt and accurate diagnosis. This case contributes to the growing body of literature suggesting that pseudoaneurysms should be considered in the differential diagnosis of new breast masses postbiopsy, particularly in settings of abnormal or unexpected clinical or imaging findings. Our experience underscores the effectiveness of ultrasound-guided thrombin injection as a safe and efficacious treatment modality, providing a valuable reference for clinicians faced with similar challenges. It also highlights the importance of a multidisciplinary approach in managing complex cases, ensuring comprehensive care that addresses both the oncologic and procedural aspects of breast disease management.

## Patient consent

Written, informed consent was obtained from the patient for the publication of this case report. The patient has reviewed the content and has agreed to the publication of their clinical information and images.
